# Global Inequality in Maternal Health Care Service Utilization: Implications for Sustainable Development Goals

**DOI:** 10.1089/heq.2018.0082

**Published:** 2019-04-26

**Authors:** Sanni Yaya, Bishwajit Ghose

**Affiliations:** School of International Development and Global Studies, Faculty of Social Sciences, University of Ottawa, Ottawa, Canada.

**Keywords:** antenatal care, skilled-birth assistance, health inequality, sustainable development goals

## Abstract

**Purpose:** Globally, low-middle-income countries continue to account for almost all of the pregnancy-related mortalities that are largely preventable through adequate utilization of essential maternal health care services such as antenatal care (ANC) and skilled birth assistance (SBA). Promoting the use of ANC and SBA services are hindered by numerous policy- and capacity-related barriers along with widespread inequality in utilization of the existing services that further exacerbates the scenario. In an attempt to better understand the geography of inequality in service utilization, we conducted this brief descriptive study by using World Health Organization (WHO) data on ANC and SBA utilization among the member states.

**Methods:** This was a descriptive study based on open access data on ANC and SBA use between 2012 and 2015 available through the Global Health Observatory of WHO. Country-level data were collected for Asia (41 countries), Africa (35 countries), Europe (35 countries), North America (10 countries), Latin America and the Caribbean (25 countries), and Oceania (16 countries). Cross-country and continent comparisons were made using dot and bar charts.

**Results:** The overall prevalence of ANC and SBA use were, respectively, 78.17% and 88.33%. Considerable disparities were found in terms of ANC and SBA use across the continents, especially in Asia and Africa. Globally, the poorest performing countries included Afghanistan, Somalia, and South Sudan where more than three-quarters of the women remain deprived of ANC and SBA services during the period of 2012 and 2015. The greatest inequality in ANC use was observed in Africa (9.4% in Somalia and 99.9% in Libya), whereas that of SBA use was observed in Asia (17.8% in Afghanistan vs. 100% in Bahrain). Europe was the most equal of all regions in terms of both ANC (66.8% in Albania vs. 99.7% in Belarus) and SBA (94.4% in Denmark vs. 100% in Lithuania) use.

**Conclusion:** Although in the majority of countries more than three-quarters of the women receive ANC and SBA services, the extent of intraregional inequality remains overwhelming especially for Asia and Africa. Progress toward maternal health-related targets should be interpreted in terms of the disparities to ensure a more even and sustainable outcome at both national and global level.

## Introduction

Equitable provision of maternal health care services such as antenatal care (ANC) and skilled birth assistance (SBA) comprise a critical component of the entire health system owing to its pivotal role in ensuring safe motherhood and survival and health of newborns as well the overall well-being of families and communities.^1–3^ Maternal mortality is a leading cause of mortality among women of reproductive age with the prevalence being significantly pronounced in low-resource settings. During past few decades, especially since the 1990s (launching year of the millennium development goals [MDGs]), the global community has witnessed massive undertakings to reverse the situation and also seen unprecedented declines in maternal and child mortality rates.^4^ However, post-MDGs estimation reveals that the progress remains largely uneven across and within regions as high rates of pregnancy-related mortalities and non-/underutilization of essential maternal health care services continue to be disproportionately higher among the most marginalized communities.^5–7^ The widening socioeconomic gradient in maternal health care utilization is a growing concern for low-middle-income countries (LMICs) in pursuit of achieving the sustainable development goals (SDGs).^7–9^

Current statistics suggest that in high-income countries very few women die from childbirth, and the key maternal health care services, for example, antenatal and professional child birth services are nearly universal.^10,11^ In contrast, LMICs account for ∼99% of global maternal mortality^12^ with the coverage of SBA being as low as 50% (in sub-Saharan Africa).^13^ The main challenges for promoting maternal health care utilization in resource-limited settings are surrounding those of accessibility and affordability barriers stemming from various infrastructural,^14^ skilled human resource,^15^ technological^16^ and financial^17^ issues at health systems level and poor health literacy,^18^ self-efficacy,^19^ and behavioral^20^ factors at the individual level. Although much has been achieved in terms of reducing the structural barriers to utilization of care, much remains to be done. With the expiration of MDGs, further initiatives are being taken (e.g., SDGs) toward the goals of promoting maternal and child health through an enhanced focus on addressing inequality in utilization of obstetric care and professional delivery services.

Experience gained from the MDGs provides the basis for realizing that health care inequality is a multidimensional issue that undercuts all development efforts and delays progress.^8,21–24^ This is particularly the case for maternal health care utilization in the LMICs owing to women's inferior socioeconomic status^25,26^ and inadequate opportunities for empowerment.^4,27^ Therefore, addressing inequality in maternal health care, including SBA, is an overarching priority, particularly in the countries that failed to meet the MDGs. Health sectors in both HICs (high-income countries/countries with minimum per capita GNI of $12,056, as per World Bank) and LMICs need to make strategic approaches to ensure that women's access to the core maternal health care services is not conditional on their socioeconomic status and pay special attention to the marginalized population who are often unable to uptake services even when no fees are incurred.^28^ The challenge is clearly of significant magnitude and would require huge input in financial and logistical terms accompanied by firm commitments for action. Addressing health inequality is also reliant on generating and provision of high-quality data to facilitate evidence-based actions and monitoring the progress at national and international levels. We undertook this study based on open-access data as an exemplary effort to show the relative inequality in ANC and SBA prevalence across and within the six continents. Through the descriptive analysis and interpretation in the context of SDGs, this article serves the purpose of informing the actors at local and international level regarding this increasingly relevant issue that deserves more intense research and policy attention.

## Methods

### Source of data

Data for this study were extracted from Global Health Observatory (GHO), which is popularly known as the gateway of World Health Organization (WHO) to population health data. GHO database is dedicated to providing most recent data on a wide range of health indicators for the WHO member states, including MDGs, infectious diseases, maternal and child nutrition, reproductive health, and health care utilization. The database has been growing in popularity given the user-friendly nature of the system and reliability of the data, which makes it a crucial resource of information for health researchers especially in the developing countries. Data are gathered from a multitude of sources such as national birth and death registration databases, demographic and health surveys, and research projects and publications.^29^ For this study, we extracted data on two indicators relevant to maternal health care utilization, for example, ANC, and SBA. Years of data collection ranged from 2012 to 2015 and for some countries data were available on ANC only but not SBA, and vice versa.

### Data analysis

This was a descriptive study and data analysis involved graphical presentation of the prevalence of utilizing ANC and SBA services. WHO defines ANC as the health services provided by skilled health care professionals to pregnant women and adolescent girls along the continuum of care risk identification, prevention, and management of pregnancy-related diseases, and health education and health promotion.^30^ An SBA is defined as an accredited health professional (e.g., midwife, doctor, and nurse) having the skills necessary for managing normal (uncomplicated) pregnancies, childbirth and the immediate postnatal period, and in the identification, management, and referral of complications in women and newborns.^31^

Countrywise percentages of ANC and SBA use were plotted side by side for easier comparison using dot and bar plots. Owing to space constraints and better interpretability, we grouped the countries into six regions: Asia, Africa, Europe, Latin America and the Caribbean, North America, and Oceania. Countries with higher and lower than global average of ANC and SBA use were presented as map charts. All charts were prepared with R studio.

## Results

**FIG. 1.**
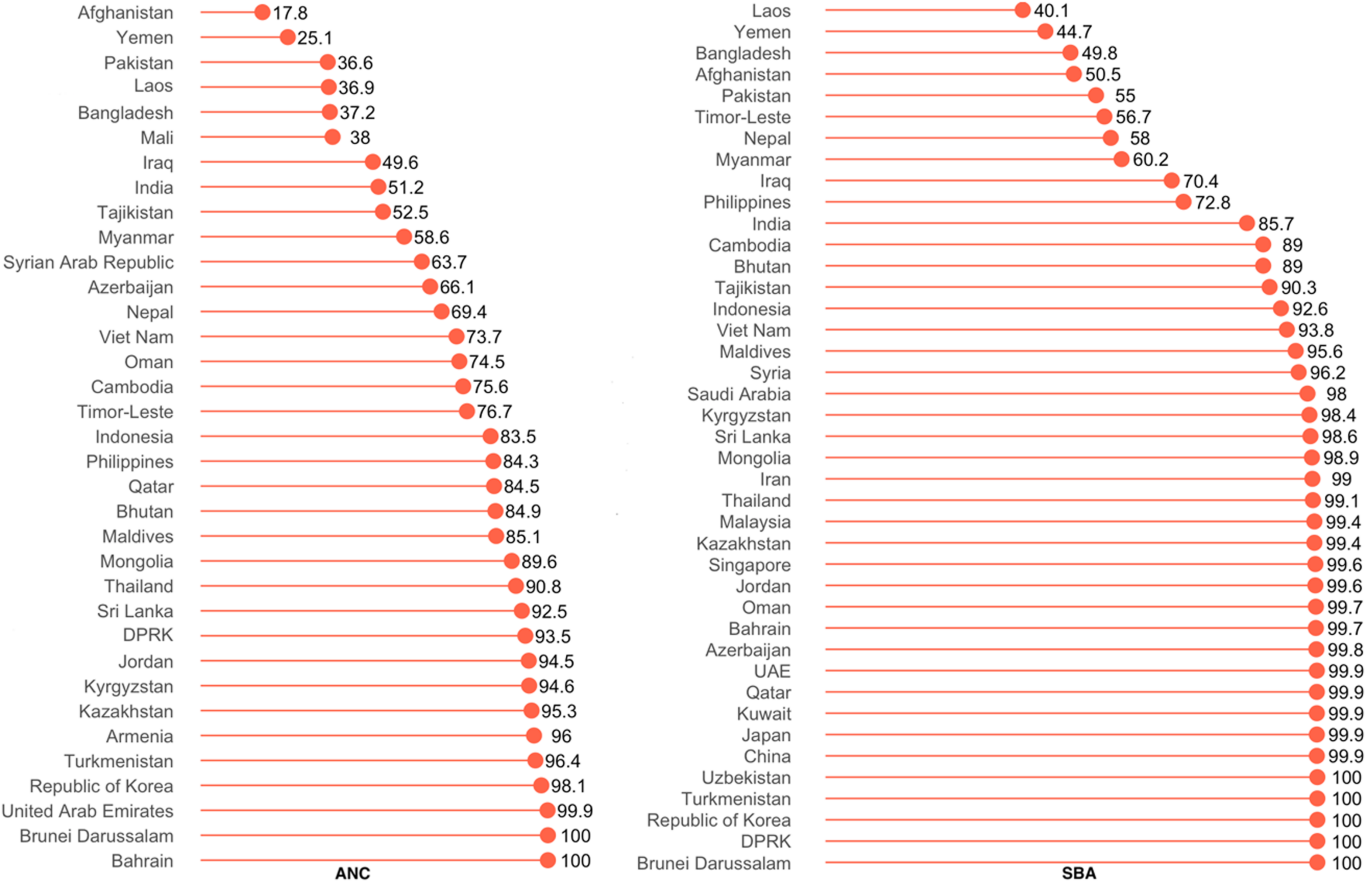
Prevalence of utilizing ANC and SBA services in Asia. ANC, antenatal care; SBA, skilled birth assistance.

Globally, the overall prevalence of ANC and SBA use were, respectively, 78.17% and 88.33%. The prevalence of ANC use among Asian countries was lowest in Afghanistan (17.8%) and that of SBA was lowest in Laos (Fig. 1). ANC and SBA were universal in several countries, including Brunei, Bahrain, UAE, South Korea, and the Democratic Republic of Korea. In general, the prevalence of ANC use was higher than that of SBA.

**FIG. 2.**
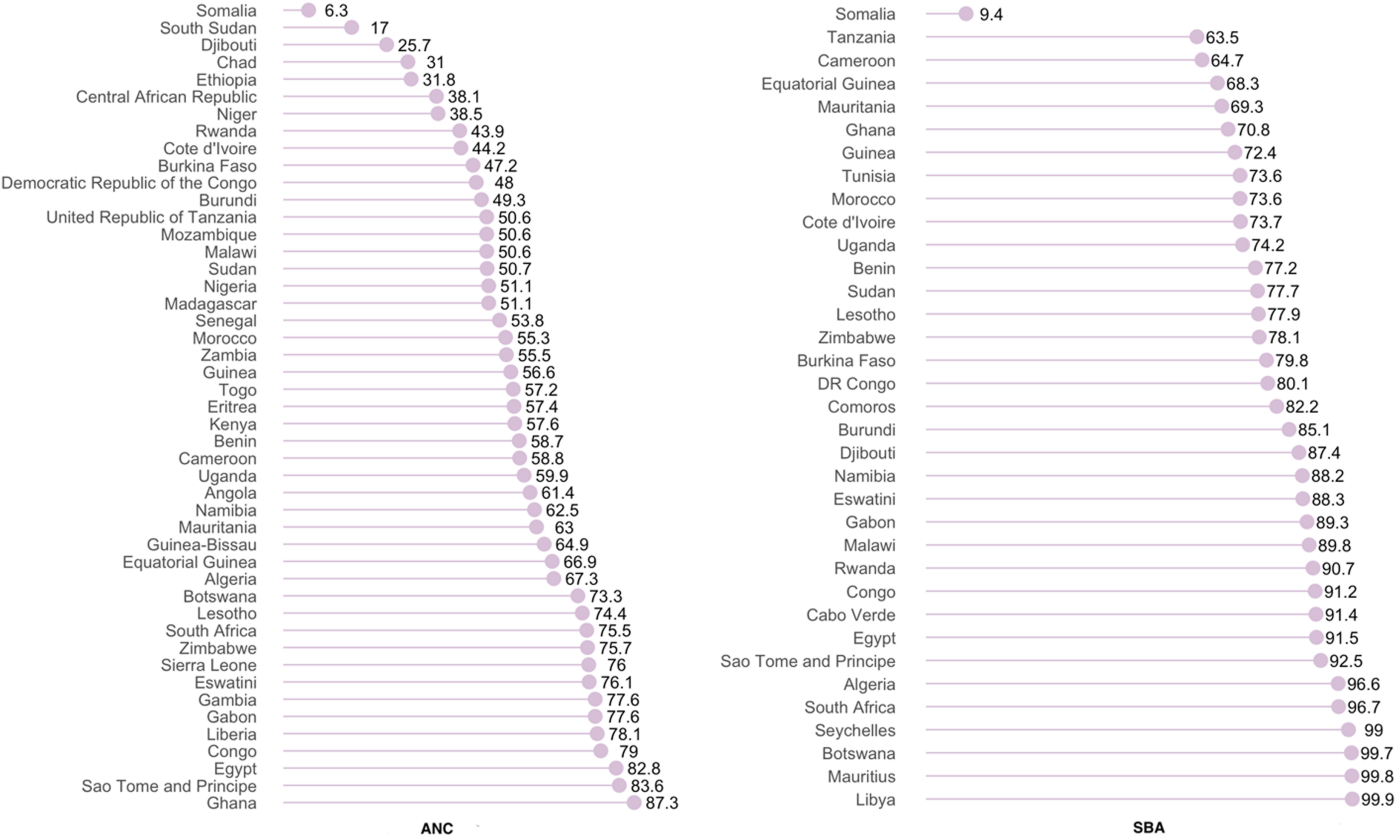
Prevalence of utilizing ANC and SBA services in Africa.

In Africa, the lowest prevalence of ANC and SBA use was observed in Somalia: 6.3% and 9.4%, respectively (Fig. 2). ANC and SBA were universal in several countries, including Brunei, Bahrain, UAE, South Korea, and Democratic Republic of Korea. In general, the prevalence of ANC use was higher than that of SBA. None of the countries has universal use of ANC, whereas SBA was near universal in Seychelles, Botswana, Mauritius, and Libya.

**FIG. 3.**
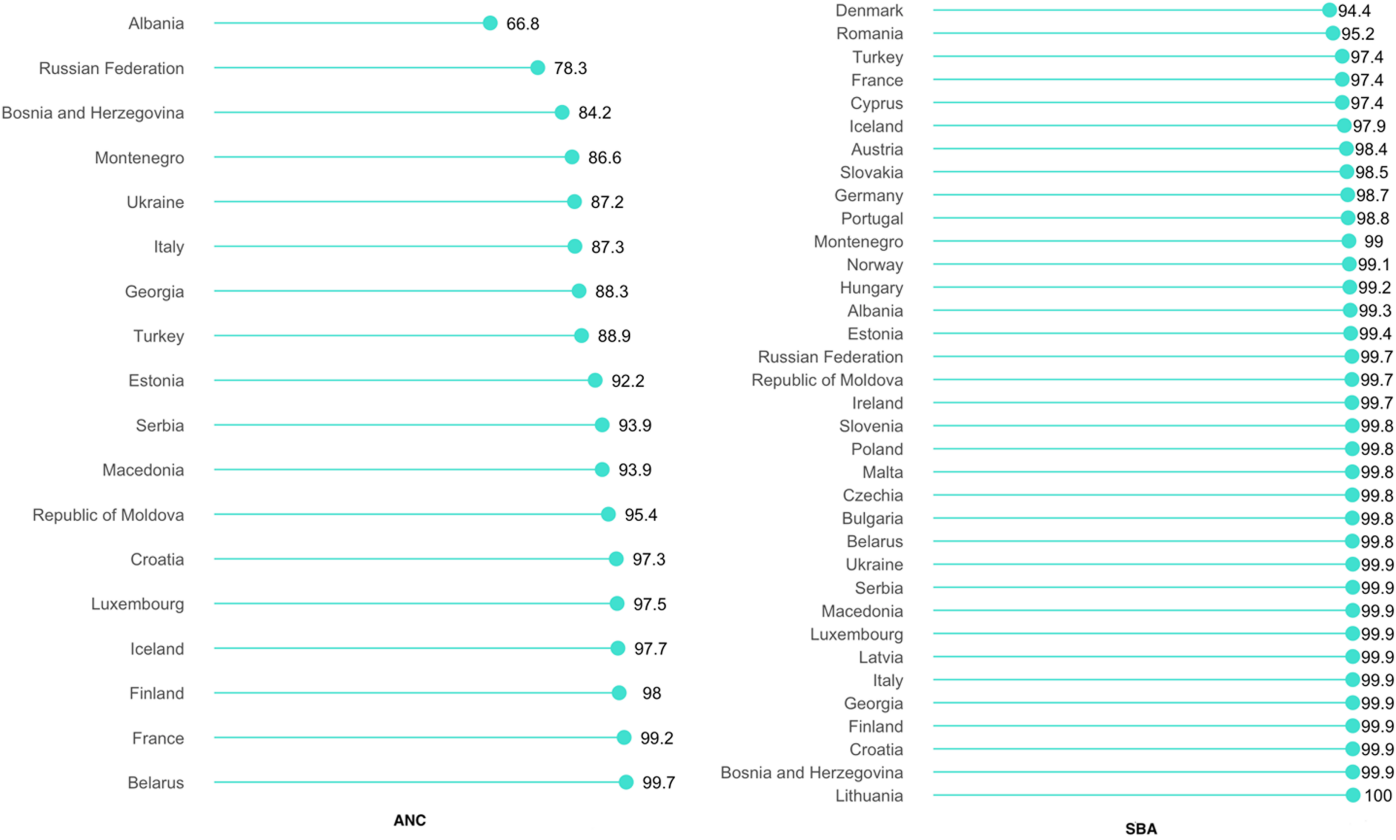
Prevalence of utilizing ANC and SBA services in Europe.

As shown in Figure 3, the prevalence of ANC use ranged from 66.8% in Albania to 99.7% in Belarus, whereas that of SBA was near universal in almost all of the countries. As was the case for Asia and Africa, the average prevalence of SBA use was higher than that of ANC use.

**FIG. 4.**
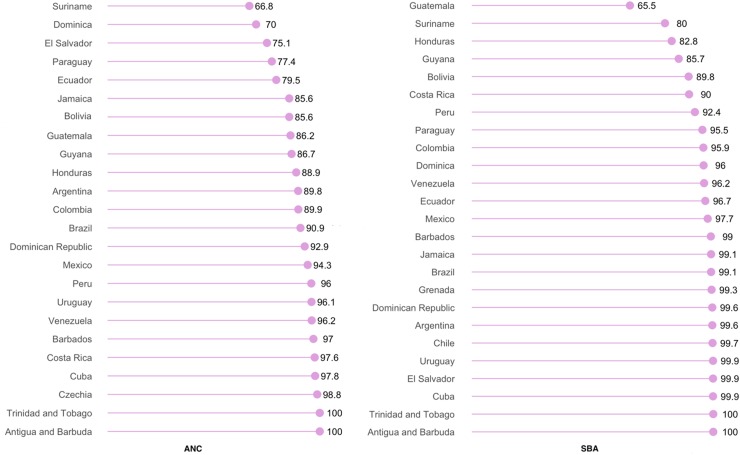
Prevalence of utilizing ANC and SBA services in Latin America and the Caribbean.

Among the countries in Latin America and the Caribbean, the lowest prevalence of ANC use was observed for Suriname (66.8%), and that of SBA use in Guatemala (65.5%). ANC and SBA use were universal in Antigua and Trinidad (Fig. 4). In majority of the countries, use of ANC services was more prevalent than that of SBA use.

**FIG. 5.**
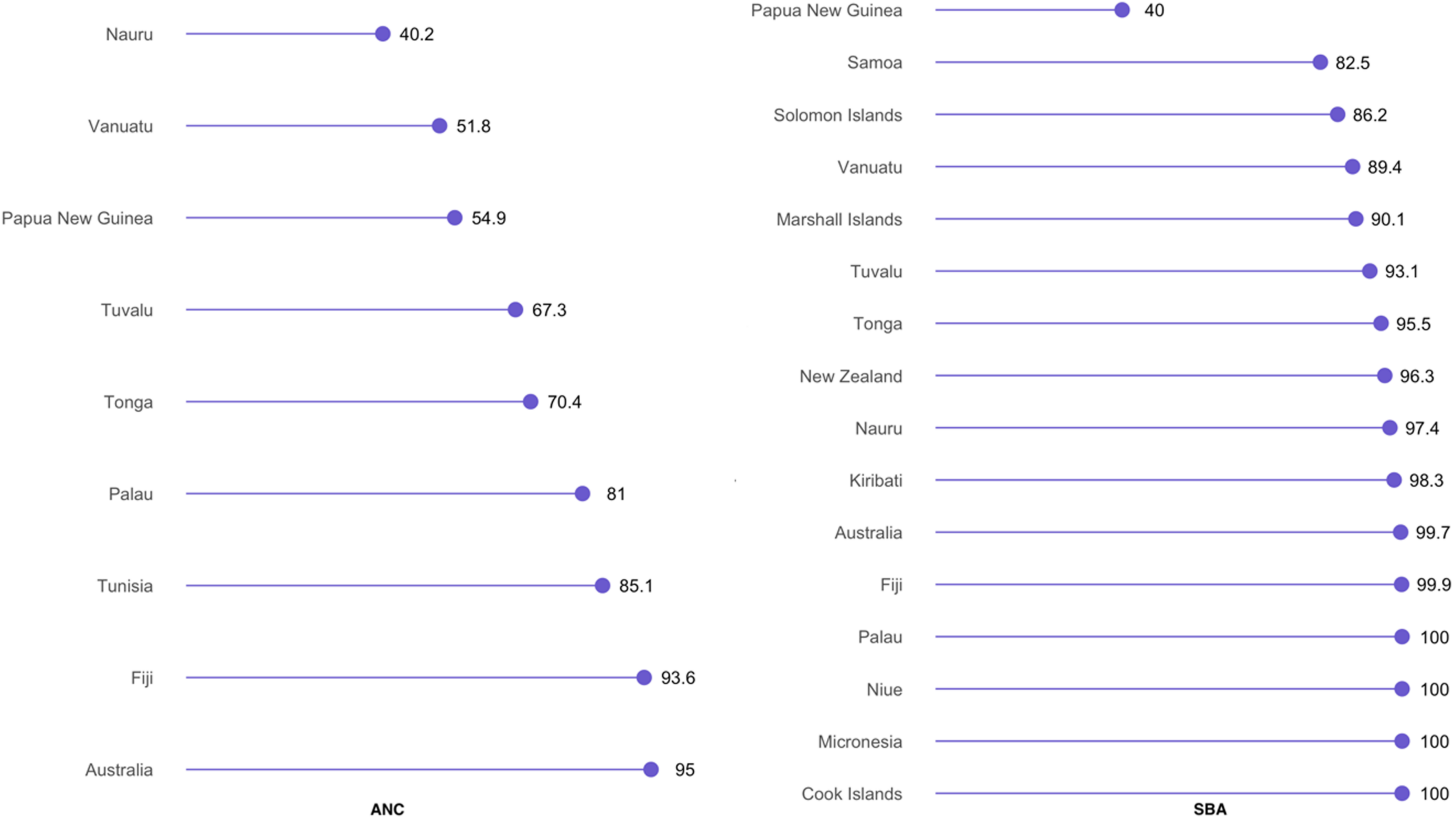
Prevalence of utilizing ANC and SBA services in Oceania.

In Oceania, the prevalence of ANC was highest in Australia (95%) and lowest in Nauru (40.2%). In contrast, SBA use was lowest in Papua New Guinea (40%), and was universal in Palau, Niue, Micronesia, and Cook Islands (Fig. 5).

**FIG. 6.**
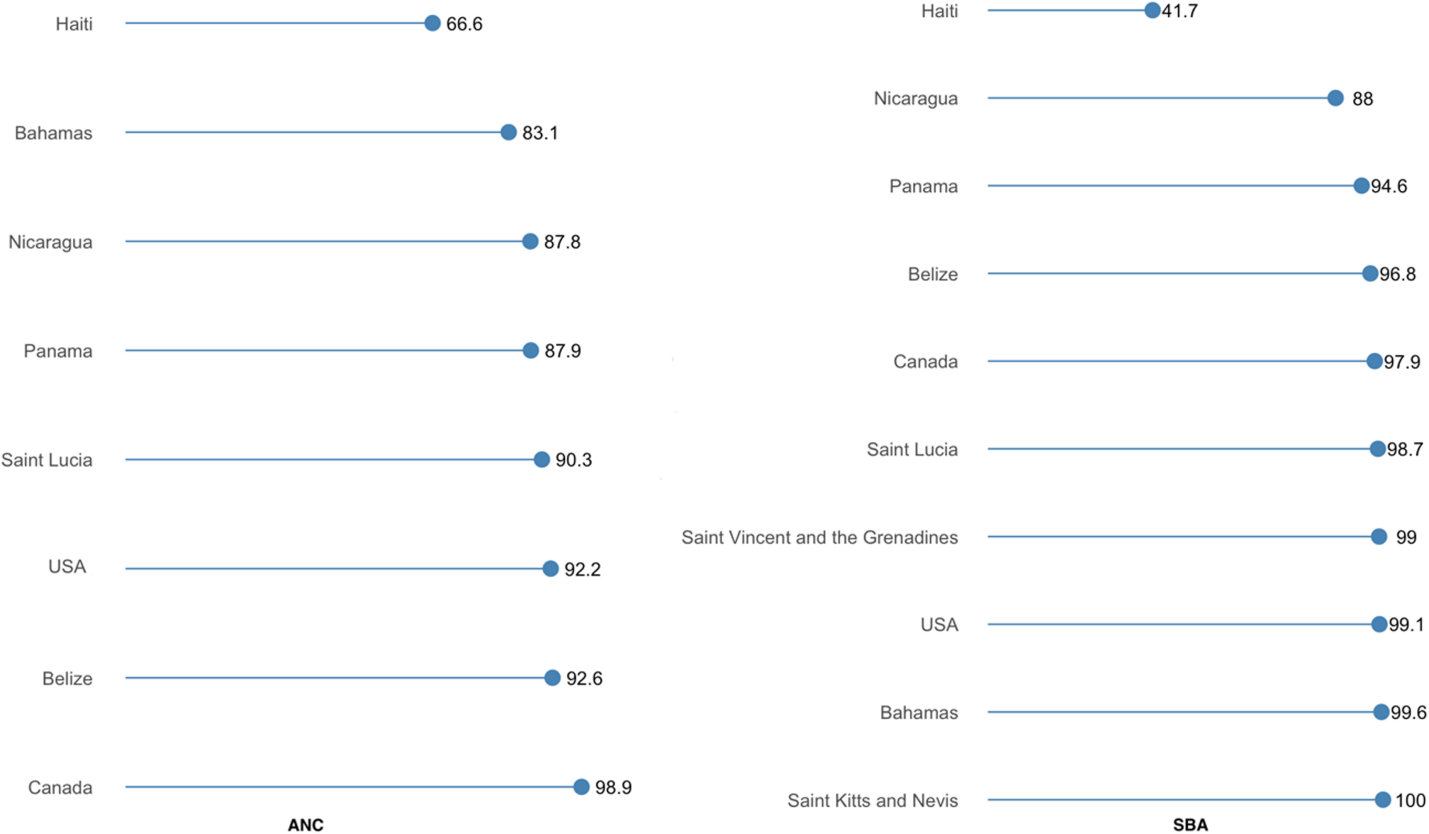
Prevalence of utilizing ANC and SBA services in North America.

Figure 6 shows that among the North American nations, Haiti had the lowest prevalence of both ANC and SBA use, and was the country where the prevalence of ANC and SBA use differed most markedly (66.6% vs. 41.7%). ANC and SBA services were near universal in Canada.

**FIG. 7.**
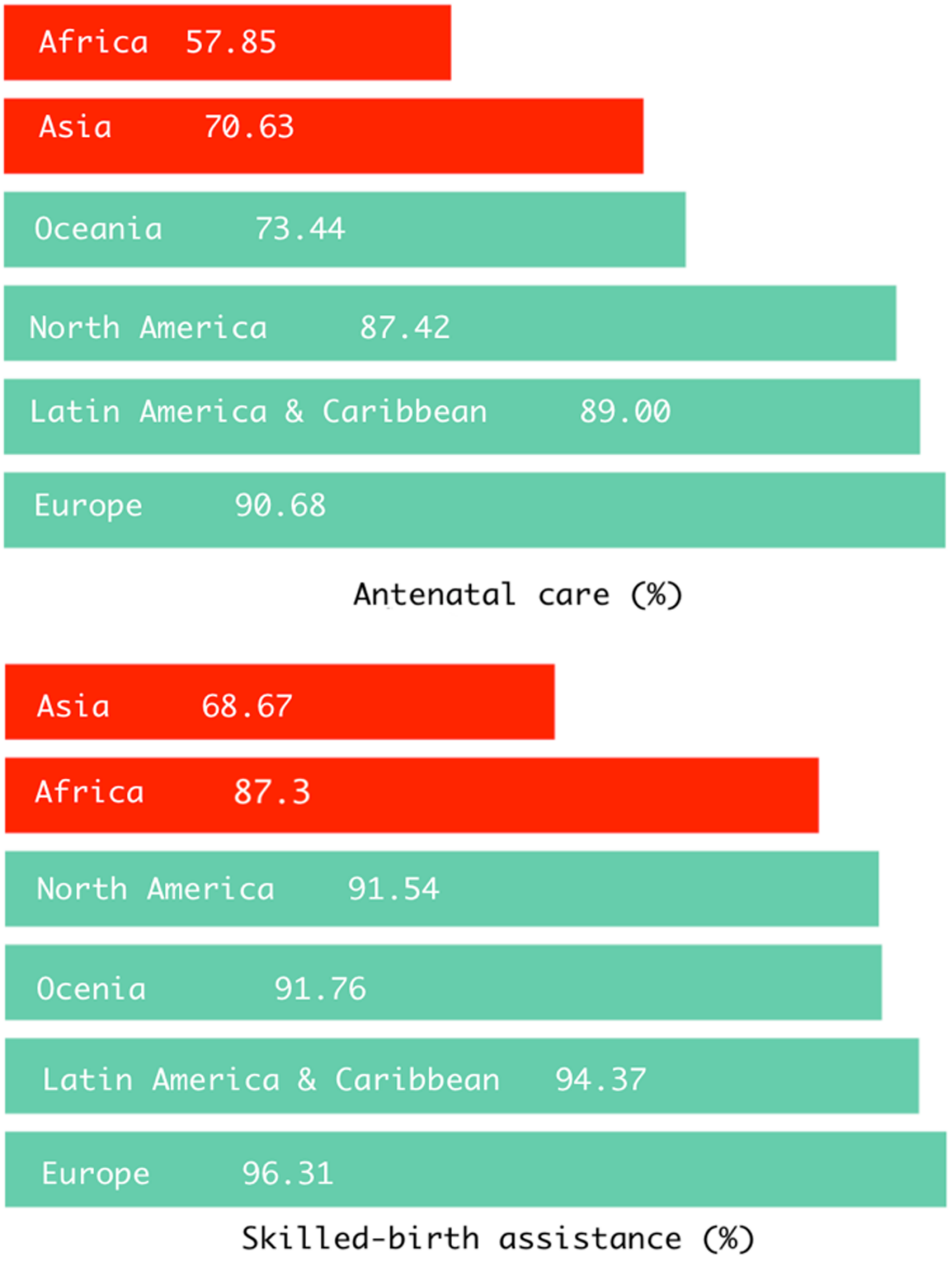
Regional inequality in the utilization of ANC and SBA services.

Figure 7 indicates that Europe as a region had the highest prevalence of ANC and SBA use followed by Latin America and the Caribbean. Overall, the prevalence of ANC use was lowest in Asia and that of SBA was lowest in Africa. The overall prevalence of SBA use was higher than that of ANC in all continents except North America.

**FIG. 8.**
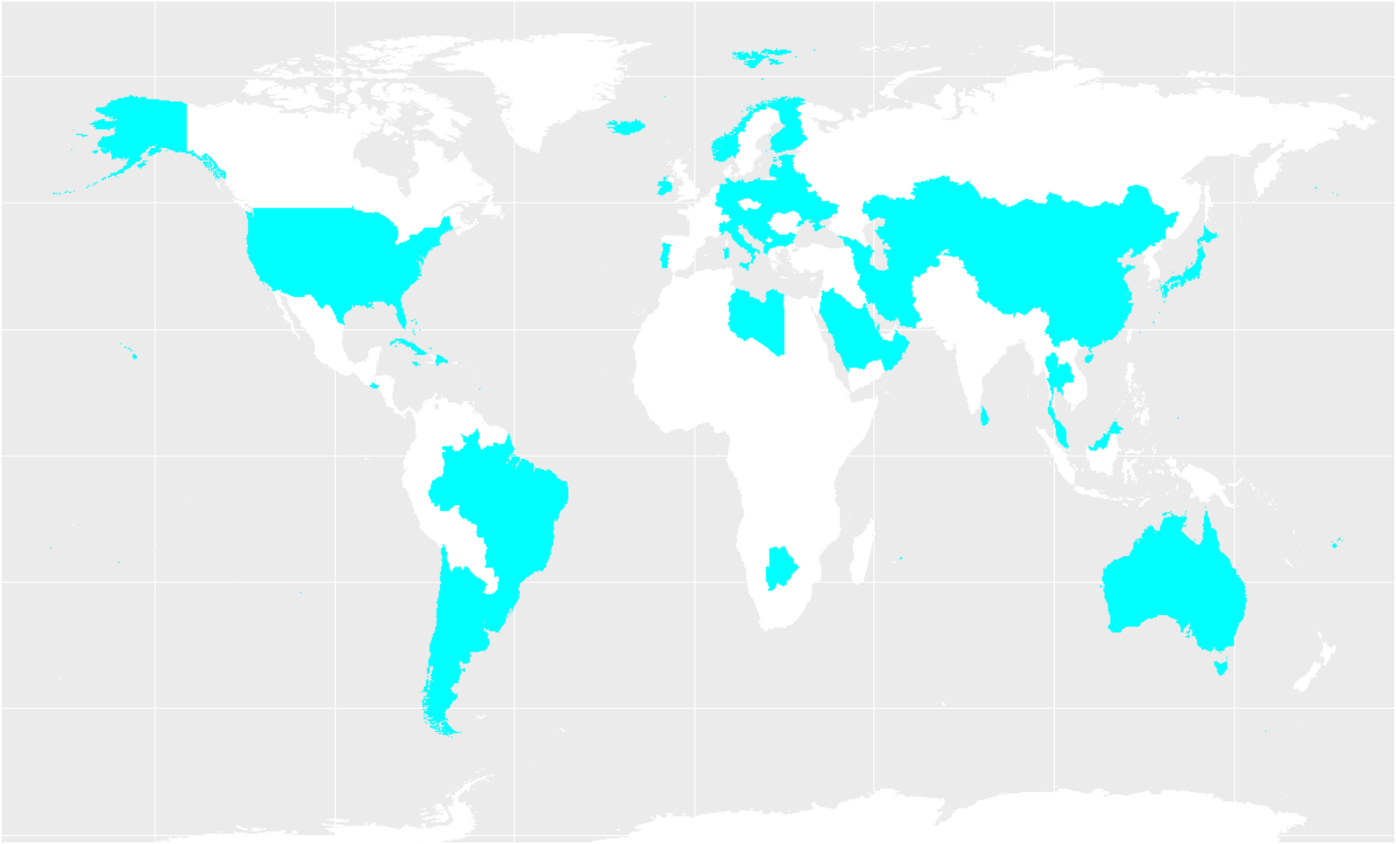
Regions with prevalence of ANC and SBA use above global average.

**FIG. 9.**
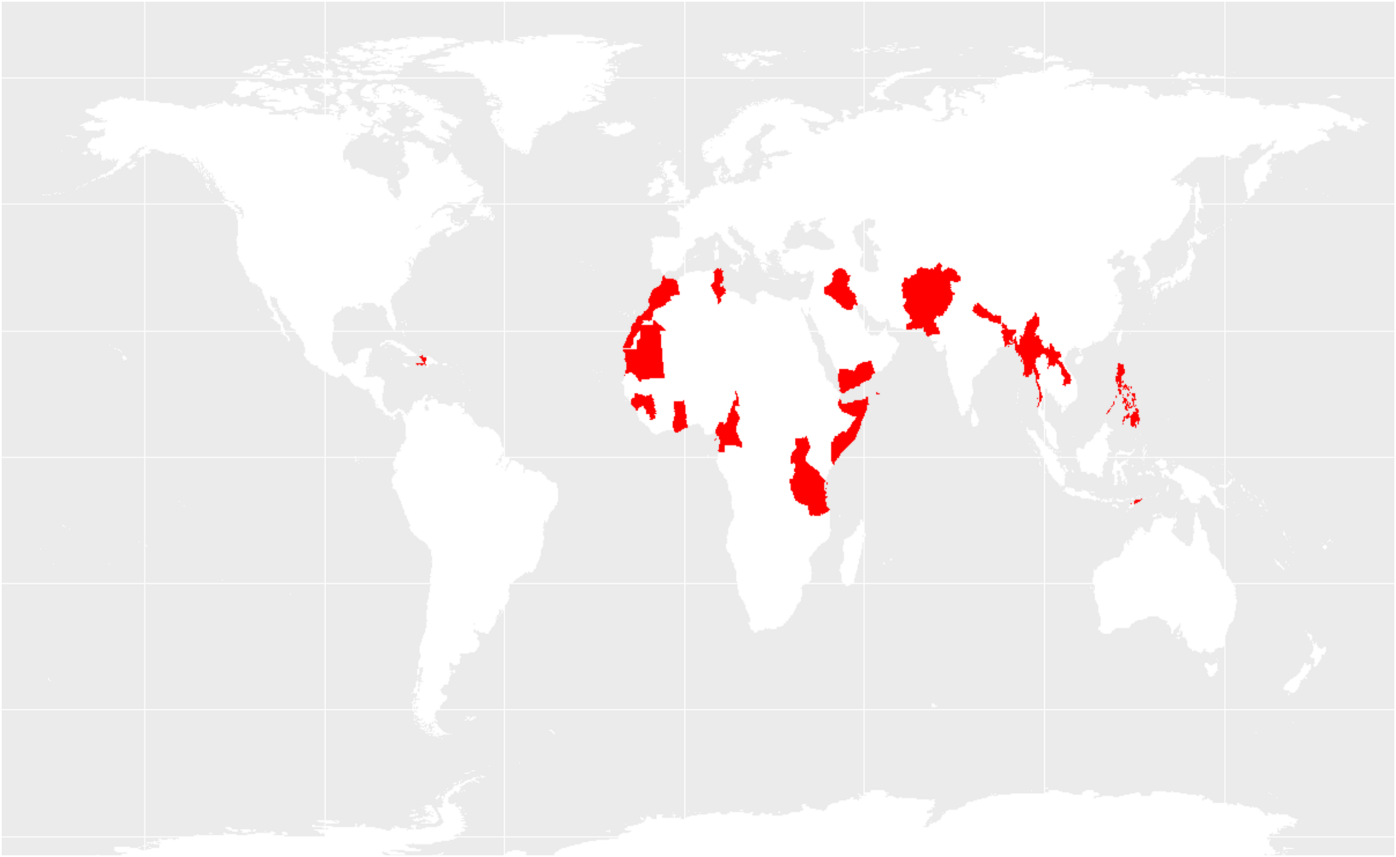
Regions with prevalence of ANC and SBA use below global average.

Countries with ANC and SBA coverage above and below global average are presented in Figures 8 and 9, respectively. The majority of the countries that rank below global average fall within the Asian and African region with a few exceptions in Latin America and Oceania.

## Discussion

Several interesting findings emerged from the analysis that are worthy of notice. First, the prevalence of ANC use was comparatively lower than SBA. This pattern was observed for both high-middle-income countries and LMIC. At the continental level, however, only North America had a higher prevalence of ANC use than SBA. The difference between the prevalence of ANC and SBA use was most remarkable for Africa—57.85% ANC versus 87.3% SBA. Inadequate-/nonuse of ANC services reduces the opportunities for detecting and addressing the diverse complications that may arise for the mother (hypertension, diabetes, and miscarriage)^32,33^ as well as for fetal surveillance that is crucial for vital examinations such as intrauterine growth retardation.^34,35^ Furthermore, women who do not attend ANC are also less likely to use professional childbirth services and thus altogether bear an increased risk of poor pregnancy outcomes.

Second, the interregional disparity was also more pronounced for ANC use. For instance, among the regions with above average prevalence of ANC and SBA use, the prevalence of ANC ranged from 73.44% in Oceania to 90.68% in Europe. In contrast, that of SBA ranged from 96.31% in Europe and 91.54% in North America. Countries with suboptimal ANC prevalence should, therefore, be given special emphasis for policy and action to enable them to be on track for meeting the maternal and child health-related SDGs.

Third, inequality in the use of both ANC and SBA was most noticeable among countries in Africa and Asia. These are also the regions where the prevalence of both of the indicators was lower than the global average. In majority of the countries in Asia, ANC services are used by more than three-quarter of the women and used almost universally in several countries. In Africa, in contrast, although in majority of the countries more than three-quarter of the births are assisted by a skilled professional and is near universal in some countries, only a handful of the countries had an ANC prevalence of 75% and higher, but was universal in none. In general, the prevalence of both ANC and SBA use was lower for African countries compared with their Asian countries of similar per capita GDP. For instance, Botswana, South Africa, and Namibia had a lower prevalence of ANC use compared with Asian economies having similar or lower per capita GDP such as Thailand, Sri Lanka, and Bhutan. This is an indication that coverage of ANC at national level is less sensitive to the measures of income in Africa than in Asia. The variation might be accounted for by differences in national macroeconomic objectives as well as health and disease control priorities in these two regions. For this analysis, we did not have data on intracountry disparities on ANC and SBA use; nonetheless, it is assumable that at national level the situation could be far worse among the disadvantaged communities and especially among poorest of the poor.

Last but not least, intercountry disparities were vivid in all the continents especially in Asia and Africa. With nearly four-fifth of the women receiving ANC and SBA services, the overall scenario of ANC and SBA use may seem appropriate at global scale. The implementation period of MDGs has brought about an unprecedented decline in global maternal and child mortality rates. Despite the remarkable progress, high maternal mortality rates continue to persist especially across Asia and Africa where a large proportion of the women do not avail essential maternal health care services such as ANC and SBA. Hence, progress achieved at global level may overshadow the widening inequalities within certain countries that are failing to keep up with the internationally set goals. Unequal progress toward the maternal health care-related MDGs suggests that overall success of the efforts would ultimately rely on eliminating the disparities at all levels. Ensuring progress in an equitable and sustainable manner is, therefore, a necessary imperative that the health policy makers need to prioritize, particularly in the countries that are lagging behind the targets regarding maternal health care.

One distinguishing feature of SDGs in relation to MDGs is its global scope aiming to enhance population health, environment, and development in all countries across the development spectrum. Not surprisingly, in both of the approaches maternal and child health-related goals occupy a pivotal position as improvement in these indicators is crucial to ensuring the overall success of the agenda. Reducing the global maternal mortality ratio to <70/100,000 by 2030 and ensuring universal access to sexual and reproductive health care represent two hallmark objectives of SDGs. From the perspective of SDGs, the goals 3 (promoting health and well-being), 5 (gender equality and women's empowerment), and 10 (reducing inequality within and across countries) are directly relevant to maternal and health child health and health care, and failure to prioritize these areas can significantly halt overall success in the LMICs (countries with per capita GNI of $996 to $3895, as per World Bank). Evidence suggests that access to quality maternal health care has significant implications on quality of life and well-being among mothers^36,37^ which serve as the prerequisite of women's socioeconomic empowerment^38,39^ and promoting gender equality.^40,41^

In this study, we took advantage of these publicly available data from WHO to explore the situation of inequality in ANC and SBA use across and within continents. Studies of this kind are particularly important as they can inform health professionals and policy makers and facilitate evidence-based interventions to combat inequality at local and global stage. To date, a few comparative analyses of maternal health care have been carried out; however, none has focused on the inequality within and across continents from the perspective of SDGs. Therefore, in this study we presented the WHO data in a more informative manner to better understand the geography of inequality in maternal health care utilization. One important limitation of the study was that we were unable to show the intracountry sociodemographic inequalities due to lack of data. The first step to addressing the inequalities would require identifying the at-risk population through conducting large-scale population surveys to facilitate informed decision making. Future studies should focus on exploring the social and political determinants that underlie the differential progress toward the use of maternal health care services.

## Conclusion

Apart from the universal use of ANC and SBA in the high-income countries, overwhelming disparities exist in a large number of countries across Asia and Africa. To promote equality in achieving the maternal health and related targets, progress should be assessed in terms of the disparities at both national and global level. Maternal health care cuts across three core components of SDGs, including population health and well-being, gender equality, and reduction of inequality within and among countries. Increased international collaboration is necessary to help the low-performing countries to be on track by enhancing both the quality and coverage of maternal health care interventions.

## Ethics Statement

Data used in this study were open access and represent country level statistics only; therefore, no institutional review board approval was necessary.

## Abbreviations Used

ANC: antenatal care
GHO: Global Health Observatory
LMIC: low-middle-income countries
MDG: millennium development goal
SBA: skilled birth assistance
SDG: sustainable development goals
WHO: World Health Organization
